# Genome Alteration Print (GAP): a tool to visualize and mine complex cancer genomic profiles obtained by SNP arrays

**DOI:** 10.1186/gb-2009-10-11-r128

**Published:** 2009-11-11

**Authors:** Tatiana Popova, Elodie Manié, Dominique Stoppa-Lyonnet, Guillem Rigaill, Emmanuel Barillot, Marc Henri Stern

**Affiliations:** 1 Centre de Recherche, Institut Curie, 26 rue d'Ulm, Paris, 75248, France; 2 INSERM U830, Institut Curie, 26 rue d'Ulm, Paris, 75248, France; 3 Department of Tumor Biology, Institut Curie, 26 rue d'Ulm, Paris, 75248, France; 4 University Paris Descartes, 12 rue de l'Ecole de Médecine, Paris, 75270, France; 5 Translational Research Department, Institut Curie, 1 avenue Claude Vellefaux, Paris, 75475, France; 6 MIA 518, AgroParisTech/INRA, 16 rue Claude Bernard, Paris, 75231, France; 7 INSERM U900, Institut Curie, 26 rue d'Ulm, Paris, 75248, France; 8 Ecole des Mines ParisTech, 35 rue Saint Honoré, Fontainebleau, 77305, France

## Abstract

We describe a method for automatic detection of absolute segmental copy numbers and genotype status in complex cancer genome profiles measured with single-nucleotide polymorphism (SNP) arrays. The method is based on pattern recognition of segmented and smoothed copy number and allelic imbalance profiles. Assignments were verified by DNA indexes of primary tumors and karyotypes of cell lines. The method performs well even for poor-quality data, low tumor content, and highly rearranged tumor genomes.

## Abstract 2

GAP, a method for analyzing complex cancer genome profiles from SNP arrays, performs well even with poor quality data and rearranged genomes

## Background

Alterations of genomic DNA are hallmarks of cancer [1]. These genetic alterations include point mutations and small insertion/deletion events, translocations, copy-number changes, amplifications, and losses of heterozygosity. Chromosome copy-number alterations and homozygosities (uniparental disomies) acquired during cancer evolution are believed to be selected as the result of the loss of function of tumor-suppressor genes and the gain of function of oncogenes. Recurrent copy-number variations (CNVs) or loss of heterozygosity (LOH) are therefore critical indicators of possible localization of cancer-related genes [1]. Both recurrent regions of alteration and patterns of genomic instability contribute to tumor classification [2]. Single-nucleotide polymorphism (SNP) arrays are presently one of the most efficient technologies for the identification of such alterations [3,4]. SNP arrays simultaneously define copy-number changes and allelic imbalances (including LOH) occurring in a tumor, at high resolution and throughout the whole genome [5].

Genome-wide SNP arrays are available mainly for Affymetrix [6] and Illumina [7] platforms. On both platforms, SNP genotypes are extracted from allele-specific signal intensities after array hybridization. Arbitrarily, the two alleles are designated as A and B, and the ratio of allele-specific signal intensities (A/B, A/(A+B), and so on, depending on the method used) provides an allelic-imbalance value. Chromosomal aberrations are identified by (a) relative copy-number changes and (b) allelic imbalances. Both platforms were originally designed for high-throughput genotyping of *normal *genomes, and they require specific normalization and data-mining strategies to study alterations in *cancer *genomes [8]. Two characteristics of genetic alterations are essential to extract from SNP data: (a) breakpoints corresponding to the boundaries of the altered regions of genomic DNA, and (b) copy number and genotype status of each such alteration.

Accurate determination of breakpoints has been addressed from many aspects, starting from reduction of nonrelevant variation to optimal breakpoint counts and positioning [9-14]. As compromises between sensitivity and specificity, these methods will perform variably, depending on the specific setting used, the quality of the primary data set, and the complexity of the tumor genomes.

Determination of copy numbers and genotype status of each alteration is more complicated, and no general solution has yet been proposed. Attempts to address this question include a manual interpretation of Affymetrix 500K SNP-array results for glioblastomas presented in [15] and an automatic copy-number recognition method based on allelic imbalances for the Illumina platform, proposed in [16]. Other methods attribute relative gain, loss, or allelic-imbalance status without addressing the determination of absolute copy number and genotype (cnvPartition from Illumina, [17-23]).

Three major sources of problems complicate the estimation of genome-wide copy number in cancer cells with SNP-array technology. The first concerns the determination of the reference point for copy-number variation (the level corresponding to the unaltered status of the tumor genome), which is not trivial for aneuploid cancer genomes with unknown underlying ploidies (diploid, tetraploid, and so on). Eventually, the reference point for a near-diploid cancer genome should correspond to normal genome status: a balanced genotype (AB status) and two copies. In the case of near-tetraploid tumors, a balanced genotype (AABB) and four copies could be proposed as the reference point. Setting the correct reference point thus depends on recognition of the underlying ploidy. This issue is considered in Attiyeh and colleagues [16], in which an aneuploidy correction factor was determined based on intensity-distribution modes in regions with balanced genotypes. Gardina and co-workers [15] directly estimated the chromosome copy-number status by using theoretic allelic ratios indicative of higher ploidy levels and then inferred tumor ploidy.

The second problem arises from the frequent contamination of cancer samples by normal stromal cells. The presence of a significant proportion of normal DNA in a sample diminishes the amplitude of measured signal changes reflecting rearrangements in the tumor DNA. Any fixed threshold-based method of copy-number variation recognition may fail to distinguish the proper regions. A number of publications have addressed this issue [17,18,24]. Staaf and colleagues [17] proposed a strategy for copy-number and LOH recognition based on adjusted thresholds, inferred from their study of dilution series. A model for estimation of normal DNA inclusion on the basis of measured allelic imbalances is considered in [18]. These authors also mentioned that, in addition to negative effects, a small degree of contamination could help in distinguishing somatically acquired homozygosity from germline homozygous regions.

The third problem in mining cancer SNP-array profiles is coming from intratumoral heterogeneity [25]. Although generally arising from a single cell (monoclonal proliferation), cancer progression leads to subpopulations bearing different genomic alterations (subclones) coexisting in most tumor samples. The tumor genomic profile is thus due to (a) genomic alterations shared by all tumor cells and producing few discrete steps of gains and losses, and (b) subclonal events shared by only certain subpopulations of tumor cells and producing a number of intermediate steps in the "main" copy-number profile. CNV and LOH status of an alteration specific for subclones is generally indefinable, as the measured signal reflects the sum of unknown subclonal signals in unknown proportions. An algorithm estimating the proportion of cancer cells harboring the particular alteration event was proposed in [18] and confirmed on known genetic events from a serial dilution of cancer cells with normal matched cells.

In this article, we propose a method for segmental copy-number and genotype detection from SNP arrays that takes advantage of previous findings and addresses the aforementioned issues. This method is based on SNP-array data formalization that we have called the Genome Alteration Print (GAP). The GAP of a tumor sample summarizes segmented CNV and allelic imbalance profiles into a list of segments, characterized by two corresponding averages. GAP visualization reveals the overall genomic ploidy of tumors, pinpoints the possible normal status (reference point for gain and loss), shows the level of contamination, indicates subclones, and generally characterizes the tumor genome. The *model *GAP built on theoretic distribution of CNV and allelic imbalances provides interpretation for a tumor GAP and serves as a basis for automatic recognition of the copy number and genotype of each segment.

## Results and discussion

### Generation of complex cancer genome data sets

The 300K Illumina SNP-arrays (Human Hap300-Duo) were used to study breast cancer genomes in a series of primary breast carcinomas (40 cases) and two cell lines. This series includes basal-like carcinomas (BLCs) arising in the general population (sporadic BLCs) and in *BRCA1 *mutation carriers, who are especially predisposed to BLCs [26]. Both hereditary (in *BRCA1 *carriers) and sporadic BLCs are associated with inactivation of *BRCA1 *[27], a key protein for DNA repair [28]. Analysis of breast carcinomas by SNP-arrays is complicated by the numerous genomic rearrangements associated with these tumors [29], their high stromal cell content [30], and intratumoral heterogeneity [31].

**Figure 1:**
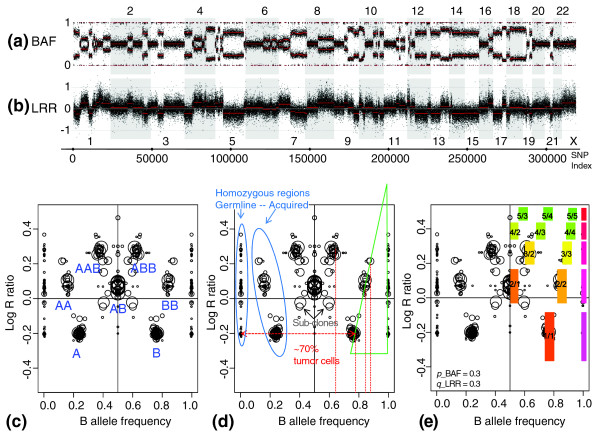
The whole-genome single-nucleotide polymorphism (SNP) array profile and genome alteration print (GAP). The whole-genome profile of genomic rearrangements in the BLC_B1_T45 sample measured by 300K Illumina SNP-array and corresponding GAP. (a) Allelic imbalances are represented by B-allele frequency (BAF). (b) Copy-number variation profile is represented by log R ratio (LRR), centered at zero. (c) The GAP of the sample is a combined sideview projection of segmented LRR and BAF. Each region of the genome is represented by two symmetric circles in the case of allelic imbalance and by one circle centered at BAF = 0.5 in the case of a balanced genotype. Attribution of copy numbers and genotypes corresponds to a near-diploid model of rearrangements. (d) "Reading" GAP pattern: the degree of stromal contamination, acquired and germline homozygosities, and subclones are indicated. (e) The best-fitting model GAP allows interpretation of the cluster structure and estimates contamination by normal DNA and contraction of the pattern on the LRR scale. Clusters are designated by the ratio of copy number to B (or major allele) counts.

Figure 1a and 1b shows the whole genome profiles of the BLC_B1_T45 sample measured on a 300K Illumina SNP-array. The copy-number variation (CNV) profile is represented by the Log R ratios (LRRs), which are the log-transformed ratios of experimental and normal reference SNP intensities, centered at zero for each sample. Allelic imbalances are represented by the B-allele frequencies (BAFs), which are the normalized proportions of the B alleles in two allele mixtures. Complexity of the profile is characterized by (a) the number of breakpoints in both profiles, and (b) the number of levels in smoothed LRRs and BAFs corresponding to the alteration states in the genomic DNA. The amplitude of both LRR and BAF changes depends on the purity of the tumor sample [17,18,24]. The main challenge is to interpret both segmental LRR and BAF values correctly in terms of *absolute *DNA copy number and LOH status, provided various amplitudes of changes, unknown underlying tumor genome ploidy, and disturbing subclonal intermediates. Specifically, DNA segments including at least 10 SNPs (~40 kb on average) were analyzed, which decreased resolution but minimized the effects of both experimental variations and short CNVs observed in population studies [32,33].

### Genome alteration print (GAP)

The method for segmental copy-number and genotypes attribution presented here is based on the structure denoted by GAP. To build the GAP, breakpoints in LRR and BAF profiles are determined separately by the circular binary segmentation (CBS) algorithm (see Materials and methods for details) [12]. Any contiguous region in both LRR and BAF profiles (region between two consecutive breakpoints from LRR and BAF breakpoints mixture) is considered to be an alteration unit (possibly unaltered) and characterized by (a) the median of LRR, (b) the modes of BAF distribution, and (c) the length of the corresponding region (in SNP counts). The list and two-dimensional visualization of all alteration units of a measured sample is denoted the GAP.

The GAP of the BLC_B1_T45 sample is shown in Figure 1c. Each alteration unit is represented by a circle, with the center coordinates equal to its BAF (x-axis) and LRR (y-axis) smoothed values. The circle radius is scaled to the relative size of the corresponding chromosome region. In other words, the structure in Figure 1c represents a combined side-view projection of segmented and smoothed profiles of LRR and BAF shown earlier in Figure 1ab. The pattern in Figure 1c has a regular structure: circles corresponding to genomic regions with similar alteration status are assembled in clusters, forming discrete steps in their projection on the LRR scale, and symmetrically disposed on the BAF scale. As "A" and "B" allele names are set arbitrarily, the BAF profile is symmetric relative to 0.5 axis, and one alteration unit is represented by two symmetric circles away from the 0.5 axis on the BAF scale. Clusters centered at BAF = 0.5 present the genome regions with balanced (heterozygous) genotype; that is, an equal representation of both (maternal and paternal) alleles.

According to standard mining of SNP-array results, the GAP pattern shows (a) normal regions, which correspond to the balanced cluster; (b) losses, which are below the level of the balanced cluster; (c) gains, which are above this level; and (d) loss of heterozygosity without copy-number change (uniparental disomy), which are the side clusters of the reference balanced cluster (Figure 1d). The overall pattern of GAP corresponds to rearrangements in a near-diploid tumor.

The balanced cluster representing the normal status is generally not centered at zero on the LRR scale, which is set by normalization. For example, in Figure 1, the functional center (the diploid balanced cluster that represents unaltered regions) is shifted up from the formal center of the LRR profile (zero on LRR scale) because of the prevailing losses versus gains observed in the tumor.

Small germline homozygous regions, detected when more than 50 successive SNPs have a homozygous call (the 50-SNP length was set arbitrarily), form side clusters at the 0 and 1 boundaries of BAF scale. These germline homozygous regions can be easily distinguished from acquired LOH (see Figure 1d). Distances between germline and acquired homozygous clusters reflect the degree of tumor-sample purity [17]. Acquired and germline homozygosities cannot be distinguished in the case of pure tumor sample or (more often) cell line.

It is worth mentioning that (a) allelic imbalance is often treated as LOH, whereas here only single allelic genotypes (A, AA, AAA...) were considered to have an LOH status; (b) although mirrored BAF (see Materials and methods) is used for all computational evaluations, the GAP structure is shown in a complete (symmetric) view for easier association with the initial SNP-array measurement (with symmetric BAF bands).

### Influence of tumor dilution and heterogeneity on GAP pattern

Breast carcinomas frequently show a high degree of stromal contamination and heterogeneity seen on the GAP pattern (Figure 1d). The triangle-like figure formed by homozygous clusters has the following interpretation. *P*% of normal DNA adds some proportion of normal (AA, AB, or BB) signal to any measured value. However, (a) this proportion depends on the corresponding copy-number status of a region and, (b) germline homozygous regions would show a pure homozygous signal, whereas cancer homozygous regions (LOH) would show a shift caused by normal heterozygous signal addition. Cancer BAF is modeled depending on the proportion of normal DNA inclusion (*p*) as the weighted sum of B-allele counts in cancer and normal genotypes related to maximal possible B allele counts at current copy-number level (see Materials and methods). For example, the calculated level of normal stromal DNA in the BLC_B1_T45 sample is approximately 30%. Such BAF dynamics also were illustrated by Nancarrow and colleagues [24] by using computer simulations. The clear linear relation between the measured mirrored BAF (mBAF) and the level of contamination by normal tissue of the tumor sample was demonstrated in [17] in dilution series.

The few isolated circles situated between one- and two-copy levels in Figure 1d could be attributed to losses occurring only in a fraction of the tumor cells (subclones). Following the logic of [18] and using our model of BAF, the proportion of cancer cells harboring this event is approximately 26%. More complicated subclonal mixtures could produce various intermediates in LRR and BAF scales.

The dynamics of change in LRR scale depend on numerous uncontrolled factors and show a high degree of variation from sample to sample. The significant dilution of a cancer sample by normal DNA clearly decreases the contrast (the amplitude of change in LRR corresponding to a copy-number change) [17]. However, universal linear dependence between LRR and contamination, similar to that for BAF, has not yet been described. The observed amplitude of LRR changes is usually smaller than expected by the initial model (log_2_(CN/2)), but the proportion between copy-number steps seems to be preserved for well-represented copy-number layers around the mean. LRR is therefore modeled by applying a simple coefficient of contraction *q *to the standard log ratio, which produces the sequence of LRR values: -*q*, 0, 0.58*q*, *q*, 1.32*q*, 2*q*,... for corresponding copy-number levels: 1, 2, 3, 4, 5,...

Model GAP that follows theoretic values for BAF and LRR with estimated contamination *p *= 0.3 and coefficient of contraction *q *= 0.3 is superimposed onto the experimental GAP of BLC_B1_T45 sample in Figure 1e.

### Diploid and tetraploid GAP patterns

**Figure 2:**
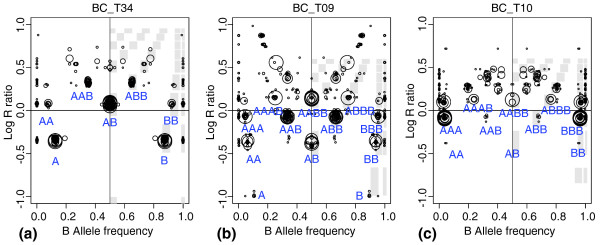
Characteristic genome alteration print (GAP) patterns. Two characteristic **(a, b) **and one unique **(c) **GAP patterns obtained in the analysis of a breast carcinoma series: **(a) **near-diploid pattern, sample BLC_ T34; **(b) **near-tetraploid pattern, sample BLC_T09; and **(c) **possible near-triploid pattern, sample BLC_T10. Attribution of genotypes is based on the type of pattern; best-fitting models are shown.

The 40 SNP-array profiles of breast carcinomas and cell lines presented two main types of GAP pattern named "near-diploid" and "near-tetraploid" patterns (Figure 2a and 2b; for more examples, see Additional data file 1). The near-diploid pattern is characterized by a single balanced cluster with one layer of losses (Figures 1 and 2a). The typical near-tetraploid pattern shown in Figure 2b has (a) two balanced modes representing a balanced heterozygous genotype on two- and four-copy levels (AB and AABB); (b) three-copy level (between balanced modes) with the full spectrum of allelic imbalances, including LOH (AAA, AAB, ABB, BBB); (c) few levels higher than four copies accounting for possible five, six, seven... copies.

The near-diploid pattern has genomic DNA mainly presented in one, two, and three copies, whereas the near-tetraploid pattern has the well-represented two-, three-, four-, and five-copy layers. These patterns appeared to be easily distinguished in the case of the high density of alteration events observed in the current series of the breast carcinomas. A unique type of GAP pattern in the series was observed in BLC_T10 (Figure 2c). This pattern is characterized by sparse balanced cluster (due to a single chromosome with balanced genotype) and very strong homozygous clusters on the 3-copy level. This may be interpreted as an almost pure triplication of a haploid genome, possibly similar to the triploid glioblastoma cases described in [15].

DNA index and karyotype were used to verify correspondence between the interpretation of GAP pattern and the actual tumor genomic status. *In silico *DNA indexes inferred from SNP arrays were very close to actual tumor DNA indexes measured with flow cytometry (FCM) analysis for 16 of the 18 breast carcinoma samples tested (Table 1, Additional data file 1). The DNA index provided by FCM characterizes DNA content of tumor genome relative to normal diploid genome, which has a DNA index defined as 1. *In silico *DNA indexes were estimated by averaging segmental copy numbers (divided by 2), inferred from the GAP pattern. For 11 cases, the difference between actual and *in silico *DNA index was less than 0.1; for five cases, it was less than 0.3. With the exception of two outliers, this difference was always less than 0.5, which is the minimal absolute error in the case of wrong assignment of the overall copy-number scale (pattern shift on +1 or -1 copy). For the two outliers (BLC_B1_T22 and BLC_T34), GAP patterns were perfectly near-diploid with a clear contrast, making cluster misattribution unlikely. The discrepancy in DNA index estimation requires further biologic verification (for example, in the case of BLC_B1_T22, there might be a pure and possibly recent duplication of the diploid tumor cells as the *in silico *DNA index was equal to half of the experimental index).

**Table 1:** Experimental and *in silico *DNA indexes and parameters of GAP model

Sample ID	DNA index FCM	DNA index GAP	DNA index OverUnder	Tumor content 1-*p*_BAF_	Contraction *q*_LRR_
BLC_B1_T14	1.14	0.85	0.98	0.85	0.37
BLC_B1_T17	0.84	0.82	0.97	0.77	0.17
BLC_B1_T19	1.6	1.63	2.93	0.4	0.27
BLC_B1_T20	1.41	1.48	3.06	0.4	0.2
BLC_B1_T22^a^	1.98	0.94	1.02	0.87	0.44
BLC_T07	1.68	1.49	3.12	0.44	0.28
BLC_T09	2.02	1.85	1.89	0.92	0.47
BLC_T10	1.88	1.9	1.07	0.95	0.47
BLC_T12	1.51	1.54	2.56	0.65	0.35
BLC_T15	1.11	0.89	0.99	0.74	0.27
BLC_T23	1.32	1.39	2.72	0.41	0.21
BLC_T31	1.91	1.84	1.48	0.84	0.45
BLC_T34^a^	1.55	0.99	1.04	0.87	0.42
BLC_T37	1.51	1.53	1.44	0.89	0.44
L_B1_T24B	1.84	1.64	2.61	0.59	0.29
L_B1_T25A	1.00	1.04	3.03	0.39	0.17
L_B1_T30	1.84	1.83	1.53	0.78	0.42
L_B1_T47	1.00	1.03	1.47	0.45	0.17

^a^Two samples with clear near-diploid pattern of GAP and discordant experimental DNA indexes.

**Figure 3:**
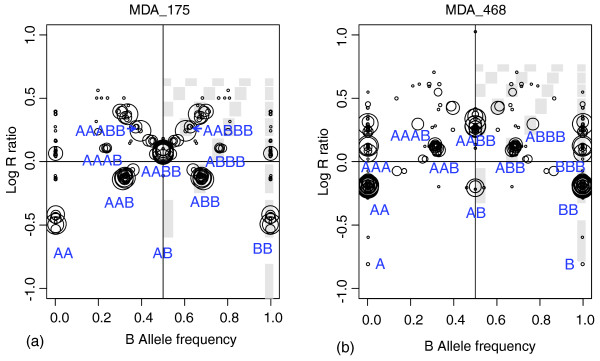
Genome alteration prints (GAPs) for breast cancer cell lines. GAPs for breast cancer cell lines: **(a) **MDA_175; and **(b) **MDA_468. Both GAPs show a near-tetraploid pattern, and genotypes were assigned accordingly.

Breast cancer cell lines with known karyotypes were used for another validation of GAP interpretation. The tetraploid breast cancer cell line MDA-MB-175-VII (MDA_175; [34]) has a clear near-tetraploid pattern of GAP (Figure 3a). The unique balanced cluster must be attributed to a four-copy level because two levels of losses visible below it could not account for 1 and 0 copies, but rather for 3 and 2 copies because of their positions and the absence of a normal contingent in the cell line. Circles on each side of the balanced cluster fit with AAAA and AAAB, and ABBB and BBBB genotypes, respectively, also implying a two-copy level. It is noteworthy that two-copy regions are represented exclusively by homozygous genotypes.

A near-tetraploid genome implies the number of chromosomes to be close to 92 (88 autosomes = two sets of diploid genomes). Copy-number summary for centromeric regions was considered a surrogate measure of chromosome number. As no SNP measurements can be performed at centromeres because of their highly repetitive DNA structure, pericentric regions were used to estimate the copy-number status of the chromosomes. The status of 39 pericentric regions (two for each of the 17 metacentric autosomes and one for each of the five acrocentric autosomes) was determined according to GAP. The number of autosomes in MDA_175 was estimated to be 86.5, which is close to the description in [34] (model number was 84 chromosomes; range, 82 to 89; verified on the cell line used for the SNP-array). Table 2 shows the frequency of occurrence of the inferred copy number of pericentric regions (also for other tumor samples considered in this study, with more-detailed information presented in Additional data file 2). A similar analysis was performed with the MDA-MB-468 (MDA_468) cell line; this hypotetraploid breast cancer cell line (modal number, 64; range, 60 to 67) [34] showed a typical tetraploid GAP pattern (Figure 3b). Estimated autosome number (71.5) matched the description, and the slight overestimation was likely due to segmental amplification in one pericentric region (Table 2 and Additional data file 2). Taken together, these results indicate correct local assignment with our approach.

**Table 2:** Frequency of inferred copy numbers at pericentric regions and deduced autosome numbers

	Copy number^a^									
Sample ID	1	2	3	4	5	6	7	8	Autosome number	Pattern^b^
MDA_175		5	9	16	4	4	1		86.5	2
MDA_468^c^		17	8	8	3	2		1	71.5	2
BLC_B1_T14	12	25	2						38	1
BLC_B1_T17	14	21	3	1					38.5	1
BLC_B1_T19		7	10	16	4	2			76.5	2
BLC_B1_T20	1	16	9	8	3	1	1		66.5	2
BLC_B1_T22	12	24	3						37.5	1
BLC_T07		15	13	8		1	1	1	70	2
BLC_T09		3	16	13		3	2	2	85.5	2
BLC_T10			21	9	2	3	3	1	87	1.5
BLC_T12		11	10	12	4	1		1	73.5	2
BLC_T15	10	27	2						40	1
BLC_T23	3	13	11	6	2			4	68.5	2
BLC_T31		5	4	25	1	2	1	1	84.5	2
BLC_T34	3	36							42	1
BLC_T37	1	16	9	6	1	4		2	70.5	2
L_B1_T24B	1	11	12	7	6	2			72	2
L_B1_T25A		37		2					46	1
L_B1_T30		3	11	23	1	1			79	2
L_B1_T47	1	34	2	1	1				47	1

^a^Frequency of inferred copy numbers (1 to 8 are indicated) at pericentric regions. ^b^1, 2, 1.5 indicates a near-diploid, near-tetraploid, and near-triploid patterns of Genome Alteration Print (GAP), respectively (Figure 2, Additional data file 1). ^c^Estimated high chromosome copy number (= 8) is likely to result from a segmental amplification in one pericentric region, leading to overestimation of the autosome number in MDA_468.

It should be noted that determination of the reference point for gain and loss attribution for complex highly rearranged cancer genomes is not always obvious, even with known patterns of rearrangements and absolute copy numbers. Samples displaying a near-diploid GAP pattern (as in Figure 2a) represent a simple situation, as their unique balanced cluster corresponding to 2-copy indicates the reference point. Near-tetraploid patterns with a unique balanced cluster at four copies (such as that of the cell line in Figure 3a) and inferred autosome numbers close to 88 indicate underlying tetraploidy, and it is logical to set the reference point to four copies in these cases. Underlying ploidy is less clear for intermediate DNA index or autosome number (between one and two, or 44 and 88, respectively), and the GAP shows a tetraploid pattern with two balanced clusters (as for BLC_B1_T19 and BLC_B1_T20 samples). Correct interpretation of gains and losses in such cases requires further biologic validation.

### Automatic recognition of segmental copy numbers and genotypes

The GAP pattern can be easily mined by automatic procedures. This procedure includes (a) recognition of a GAP pattern and (b) assignment of segmental copy numbers and genotypes to a corresponding tumor genome based on this pattern. As described earlier, the GAP is characterized by two parameters: *p*, which is the proportion of tumor contamination by normal DNA affecting BAF values, and *q*, which is a coefficient of contraction of LRR values. The automatic recognition procedure searches for parameters and position of a model GAP that best fits to the experimental GAP. Quality of fitness is assessed by genome coverage in terms of number of SNPs that are explained by the model (see Material and methods for details). In other words, the model GAP template that most closely corresponds to the experimental GAP is selected. In the second round, the model GAP is used as the basis for interpretation of the experimental GAP, and segmental copy numbers and genotypes are assigned accordingly.

The quality of pattern recognition was tested on 42 in-house samples, including the samples validated by DNA index. The procedure performed 41 correct and one erroneous recognitions, as compared with manual assessment. The problematic sample presented a high variance and low contrast, and the correct solution had a high but not the highest score. In general, the method tolerates contamination of tumor samples by normal DNA and experimental variations, as shown by correct recognition of our validated series with up to 60% of normal contamination and up to 0.17 contraction of LRR scale (see Table 1).

We considered subclones as segments located essentially between designated clusters. They could be artefacts from incorrect segmentation, or true tumor heterogeneity. An interesting case is represented by sample BLC_T31. Its first interpretation was that of a near-tetraploid pattern, but its second interpretation with a very similar score was that of a near-diploid pattern because of poor representation of the three-copy level interpreted as subclones in the latter case. The DNA index determined by FCM indicated near-tetraploidy, supporting the first interpretation (see Additional data file 1).

It should be stressed that (a) correct recognition requires good contrast between clusters and multiplicity of genetic events (for example, patterns consisting of AB and A∅ genotypes versus AABB and AA cannot be distinguished when no other evidence of a four-copy pattern exists); (b) the robustness of the quality criterion used in our method is not always satisfactory: the correct solution often differs from incorrect solutions by less than 1%; (c) the linear models used in the method diverge from experimental data in both the LRR and BAF scales when copy numbers were higher than 6-copy. However, no universal rule to correct this effect was identified on the basis of the 41 tumors examined.

### Comparative testing of GAP recognition

When characterizing rearrangements in tumor genome measured by SNP array, it is essential to extract from data (a) the degree of genomic instability displayed by the number and distribution of breakpoints, and (b) the type of each alteration. The GAP method is based on both LRR and BAF breakpoints and is therefore not directly suitable for breakpoint counting. To minimize double counting of a single breakpoint, LRR and BAF breakpoints separated by a small region (arbitrary defined as 10 SNPs) were simply merged. More complicated pooling of LRR and BAF breakpoints could allow more accurate breakpoint counting, and this would not be expected to influence the performance of the GAP method. Another way to address breakpoint detection in highly rearranged cancer genomes with possible low tumor content and noisy profiles is to use the GAP pattern as a source for secondary optimization.

The GAP method is elaborated for determination of alteration events in *complex*, highly rearranged cancer genomes (in contrast, it would be of little help for interpretation of a stable genome with few amplifications). The methods specifically developed for analysis of cancer genomes include SOMATICS [18] and BAFsegmentation [17], which reveal segments with allelic imbalances based on various models but do not produce copy numbers and genotypes. The OverUnder algorithm presented by Attiyeh and associates [16] estimates ploidy, as well as copy numbers and genotypes, and has been shown to outperform PennCNV, IlluminaCN Estimate, and CBS for the analysis of cancer genomes. We compared our automatic GAP fitting method with the OverUnder algorithm in terms of quality and consistency of recognition.

**Figure 4:**
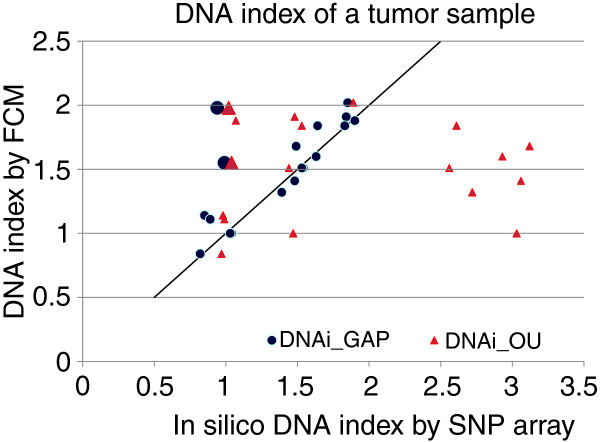
Comparison of genome alteration print (GAP) and OverUnder-based *in silico *DNA indexes with experimental DNA indexes. GAP indexes (blue circles) show excellent correspondence with experimental DNA indexes. OverUnder indexes (red triangles) show more outliers with overestimation of the DNA index. Both methods show consistent results, but not corresponding to the experimental DNA indexes (1.98 and 1.5) for two samples, designated by enlarged markers.

The OverUnder algorithm (available as Illumina Beadstudio plug-in) was initially applied to our validated series of breast carcinomas to estimate the DNA indexes (Table 1). OverUnder results for seven samples clearly deviate from experimental data (Figure 4). These samples are characterized by high levels of normal DNA contamination, as estimated by the GAP model. The GAP method tolerated normal contamination, demonstrating better overall performance.

The self-consistency of the methods was tested on the basis of dilution series available in the GEO database (GEO:GSE11976) [17]. The HCC1395/CRL2324 cell line [34] measured in this series is genetically complex and poorly defined. However, estimated copy numbers and LOH regions must be consistent for all CRL2324 samples with various proportions of tumor DNA. The results of the self-consistency test are presented in Table 3 (more details in Additional data file 3). The better self-consistency of the GAP method is obvious in terms of copy numbers and LOH. Structural reproducibility of tumor GAP pattern with various proportions of normal DNA is illustrated in Additional data file 4.

**Table 3:** Self-consistency of copy numbers and LOH in dilution series by using GAP and OverUnder analyses

GAP	CN	LOH			DNA index	Tumor DNA	1-*p *BAF	*q *LRR
CRL2324	1	1			1.45	1	1	0.45
CRL2324_79	0.93	0.98			1.46	0.79	0.8	0.35
CRL2324_50	0.9	0.97			1.44	0.5	0.42	0.22
CRL2324_47	0.78	0.96			1.49	0.47	0.42	0.24
CRL2324_45	0.81	0.96			1.5	0.45	0.35	0.18
CRL2324_34	0.69	0.93			1.53	0.34	0.27	0.16
CRL2324_30	0.73	0.93			1.52	0.3	0.25	0.12
CRL2324_23	0.72	0.93			1.59	0.23	0.26	0.14
CRL2324_21	0.7	0.92			1.64	0.21	0.14	0.12
OverUnder	CN	LOH	CN ± 1	CN CBS	DNA index	Tumor DNA		
CRL2324	1	1	1	1	2.48	1		
CRL2324_79	0.36	0.94	0.74	0.46	2.16	0.79		
CRL2324_50	0.21	0.45	0.65	0.1	2.64	0.5		
CRL2324_47	0.22	0.45	0.68	0.1	2.5	0.47		
CRL2324_45	0.34	0.45	0.71	0.11	2.85	0.45		
CRL2324_34	0.24	0.44	0.56	0.19	2.57	0.34		
CRL2324_30	0.14	0.45	0.48	0.23	2.54	0.3		
CRL2324_23	0.31	0.45	0.68	0.23	2.51	0.23		
CRL2324_21	0.07	0.47	0.12	0.05	1.11	0.21		

CN = copy number; LOH = loss of heterozygosity; tumor DNA = proportion of tumor DNA in the dilution; DNA index = *in silico *DNA index with each algorithm; 1-*p *BAF and *q *LRR are parameters of the model GAP; CN ± 1, copy numbers are considered to be consistent when the difference is less than or equal to 1; CN CBS, consistency is calculated on averaged (by median) and rounded copy-number assignments in CBS determined segments.

### GAP for Affymetrix SNP platform

Affymetrix GeneChip SNP 6.0 array was used to generate SNP profiles of the BLC_B1_T45 sample. The GAP was obtained according to the same strategy as for Illumina SNP data but by using the profile-recognition method described in [14].

**Figure 5:**
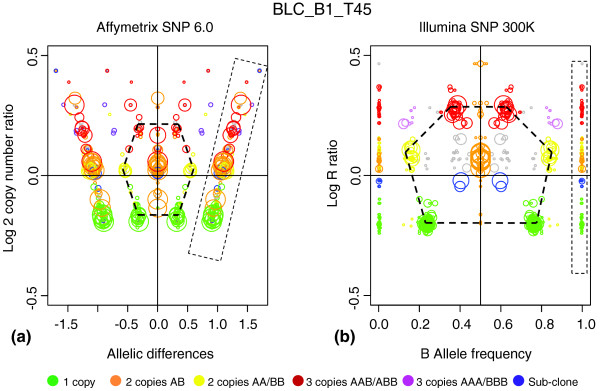
Genome alteration print (GAP) for Affymetrix single-nucleotide polymorphism (SNP) GeneChip SNP 6.0 array. BLC_B1_T45 tumor sample measured on two SNP-array platforms, analyzed by using GAP, and superimposed by color code: **(a) **GAP for Affymetrix; and **(b) **GAP for Illumina. Copy numbers obtained from the Illumina GAP were coded by colors indicated at the bottom of the Figure. Concordance between Affymetrix and Illumina patterns is illustrated by the relevant Illumina-derived color gradation on Affymetrix GAP. Germline homozygous regions are boxed. The main cluster patterns are indicated by hexagonal frames. The differences in relative cluster sizes are due to different distributions of SNPs measured along the genome in Illumina and Affymetrix chips.

Comparison of the data generated on these two platforms is shown in Figure 5. Affymetrix SNP measurements are represented by Log Copy Number Ratio and Allelic Differences as compared with Illumina LRR and BAF, respectively. Germline homozygous SNPs were omitted if fewer than 50 in a row, and are therefore represented by small clusters along two parallel lines at 0 and 1 limits of the BAF scale in an Illumina plot. Homozygous SNPs were always included in Affymetrix GAP and therefore formed large clusters represented along divergent diagonal lines (as allelic differences are dependent on copy-number levels) in the Affymetrix plot. Genome regions localized and attributed to a specific copy number in an Illumina-profiled genome were used to color code the regions in the Affymetrix SNP profile. Excellent concordance was observed between Affymetrix and Illumina patterns, as shown by relevant Illumina-derived color gradation on Affymetrix GAP. Visible differences in relative cluster sizes are due to different distributions of measured SNPs along the genome in Illumina and Affymetrix chips. The main conclusions from this comparison are (a) excellent correspondence between the two technologies in terms of copy-number variation; and (b) GAP can be used for analysis of complex cancer genomes on Affymetrix platforms.

## Conclusions

We present a method to mine complex genome alteration profiles measured with SNP-arrays. We introduce genome alteration print (GAP), a combined side-view projection of LRR and BAF segmented and smoothed profiles. The method, based on GAP pattern recognition, is fully automatic and provides segmental copy numbers and genotypes. It also estimates tumor-sample contamination by normal DNA. The method performs well, even for poor-quality data, low tumor content, and highly rearranged tumor genomes. Visualization of the GAP recognition pattern characterizes overall rearrangements in a tumor sample and can be used to verify the results. The GAP method is designed for Illumina SNP-array, but can be easily applied to Affymetrix SNP-arrays. This method could be a valuable tool to identify recurrent alterations in complex tumor-genome profiles.

## Materials and methods

### Illumina arrays

A series of 40 breast carcinomas, including cases described in [35], was analyzed, as well as the breast cancer cell lines MDA-MB-175-VII (MDA_175) and MDA-MB-468 (MDA_468) [34]. DNA was extracted from samples, and genomic profiling of the tumor samples was performed at Integragen [36] on 300K Illumina SNP-arrays (Human Hap300-Duo). SNP-array data are available through Gene Expression Omnibus [37] [GEO:GSE18799].

### Data processing

Normalization of raw data was performed with Illumina Beadstudio software version 3.3 by using standard settings (all supporting files are provided by Illumina [7]). The normalization procedure tQN proposed in [9] also was used to make BAF symmetric.

### LRR and BAF segmentation and construction of the GAP

The circular binary segmentation (CBS) algorithm (DNAcopy package, Bioconductor) [12,38] was applied to LRR and filtered BAF data separately to define breakpoints (the minimal level of significance was defined as 10^-2 ^for LRR and 10^-3 ^for BAF profiles). Smoothing of outliers was performed in both cases. LRR was smoothed by the median between breakpoints. To obtain one banded BAF profile, (a) non-informative homozygous SNPs were filtered out, based on the threshold (mBAF > 0.97), as suggested in [17]; (b) tQN normalized and reflected relative to the 0.5 axis version of BAF, named mirrored BAF (mBAF) [17], was segmented. In addition, the boundaries of germline homozygous regions, detected when more than 50 successive SNPs had a homozygous call (the number of SNPs was set arbitrarily), were included into the set of breakpoints. The mode estimation (dip-test package, [38]) was used for smoothing of the mBAF profile to maintain the contrast between balanced and slightly shifted imbalances.

Any region between two consecutive breakpoints from the LRR and BAF breakpoint mixture was considered to be an alteration unit (possibly unaltered) and characterized by (a) the averaged LRR, (b) the mode of mBAF distribution, and (c) the length of the corresponding region (in SNP counts). The list and the two-dimensional visualization of all alteration units of a measured sample were denoted the genome alteration print (GAP). For GAP visualization, each alteration unit was represented by a circle centered on BAF (x-axis) and LRR (y-axis) smoothed values, and the radius was scaled to the relative size of the corresponding chromosome region.

### Comments on stability of GAP

The CBS algorithm was used to favor sensitivity over specificity in the breakpoint-detection process, as "false" breakpoints do not significantly change the overall GAP pattern. False alteration units often appeared as artefacts at joining LRR and BAF breakpoints, but were not visible, provided the true alteration units were significantly longer. More problems were observed when the robust profile estimators were applied to poor-quality data: the absence of true breakpoints could significantly alter the GAP pattern.

### Model GAP

The model GAP was determined by the independent combination of BAF and LRR models. The BAF model was used to determine the position of clusters on the horizontal scale, and the LRR model was used to determine the *relative *position of clusters on the vertical scale.

The cancer BAF was modeled as the weighted sum of B-allele counts in cancer and normal genotypes, as a ratio of the maximal possible B allele counts at the current copy-number level:

where *p *is normal DNA proportion (and hence (1 - *p*) is tumor DNA proportion);  and  are the B and A allele counts in the tumor genotype; ( + ) is considered to be the copy-number level; and  is the B allele count in normal genome ( = 0, 1, 2). A similar model was described in [17,24]; a model proposed in [18] could also be used for GAP-method settings.

To estimate normal DNA contamination in a measured tumor sample, at least one cluster annotation (copy number and genotype) and its position on the BAF scale must be known. For example, projections of cluster centers in the experimental GAP pattern (Figure 1e) were assessed to be as follows: *BAF*^*M *^= 0.765 for the B cluster, *BAF*^*M *^= 0.845 for the BB cluster, *BAF*^*M *^= 0.885 for the BBB cluster, and *BAF*^*M *^= 0.628 for the ABB cluster. Substitution of *BAF*^*M*^, B allele counts, and copy numbers in the model provides an estimation of the contamination coefficient *p *= 0.307, 0.31, 0.309, and 0.312, respectively. As expected, inferred coefficients were very close to each other and estimated the normal DNA contamination around 30% for this sample.

The same method was used to estimate the proportion of tumor cells bearing a given rearrangement (subclone); in the case shown in Figure 1d: *BAF*^*M *^= 0.575,  = 1, + = 1,  = 1 gave the normal content estimation *p *≈ 0.74 and hence the tumor content was 1 - *p *≈ 0.26.

LRR was modeled by applying a simple coefficient of contraction *q *to the standard log ratio:; *n *is the copy number, which produces the sequence of LRR values: -*q*, 0, 0.58*q*, *q*, 1.32*q*, 2*q*,... for corresponding copy number levels: 1, 2, 3, 4, 5... The LRR of zero copy (homozygous deletion) was arbitrarily set at -3*q *(log_2_0 = - ∞, variation in real LRR is usually very large and not followed by the model).

### Fitting model GAP and copy number and genotype recognition

Automatic recognition of the tumor GAP pattern consisted of an exhaustive search for (a) the best centering of the model GAP on the LRR scale for each pair of contamination proportion (*p*) and coefficient of contraction (*q*), and (b) the best (*p, q*) couple satisfying a few necessary conditions. The genome coverage in terms of the number of SNPs explained by the model was used as the quality criterion. The necessary conditions were used to filter unusual interpretations.

GAP pattern-recognition algorithm: 1) Initiation of a grid with 0.005 cell dimension on the BAF × LRR plane and definition of the densities of alteration units in SNP counts on the grid; 2) Smoothing of the densities by averaging adjacent cells and filtering of low densities to enhance the contrast (densities were set to 0 in 95 to 98% of cells in the grid); 3) Choosing model parameters (*p, p *∈ {0,0.02, 0.04, ..., 0.86};*q*,) and setting of the GAP template with one to five copies by determining the centers and sizes of model clusters on the grid; 4) For a given pair (*p, q*), searching for the best centering of the GAP template on the grid in terms of maximal density falling into designated clusters; 5) Checking all possible combinations of *p *and *q *and ranking templates; 6) Filtering of templates according to necessary conditions. This removes from further consideration redundant interpretations with many empty clusters; 7) Choosing the best interpretation, superimposing the model GAP to the experimental one, and ascribing copy number and genotype to each alteration unit after the annotation of its closest cluster on the template.

In the case of low contrast between clusters, additional adjustments of recognition are necessary to attribute correctly the alteration units located between designated clusters. A confidence score is attributed to all alteration units (depending on the distance to the nearest model cluster(s)), and the *linear *copy number and genotype profiles are adjusted by keeping confident assignments and correcting less-confident assignments.

### Experimental estimation of ploidy and karyotyping

The DNA content of tumor samples was obtained with flow cytometry (FCM) analysis after propidium iodine staining, as described in [39]. The DNA index is equal to 1 for normal diploid cells. A karyotype of MDA_175 was obtained by a routine procedure [40].

### Estimation of DNA content and chromosome number based on SNP data

The inferred copy-number profile was averaged along the genome, providing the DNA content of the corresponding cancer sample.

Chromosome copy numbers were characterized by the status of pericentric regions, defined as the alteration units directly before or after the centromeric part of the chromosome (which has no SNP measurement *per se*). The definition of pericentric region therefore depends on the SNP chip used for genotyping. Regions less than 10 SNPs were ignored. If the pericentric alteration unit is a small region (less than 100 SNPs), setting the chromosome copy number on the basis of this alteration unit could be erroneous and could therefore interfere with karyotype assessment.

### Dilution series

The dilution series described in [17], measured by Illumina 370 K array and available in the GEO database [37] [GEO:GSE11976], were processed in a similar way to in-house tumor samples: (a) normalization by the method proposed in [9]; (b) segmentation of LRR and BAF profiles by CBS, in the same way as described in subsection 3 of the Materials and Methods; (c) construction of GAP, GAP pattern recognition, and copy-number assignment.

### Results of the OverUnder [16] algorithm

The OverUnder plug-in was applied to the data normalized in BeadStudio 3.3, with window length equal to 51. OverUnder produces continuous copy-number values, which were rounded to discrete values and then summarized in comparison tables. As rounding can introduce artificial discrepancies, the procedure was slightly modified so that copy numbers were considered to be equal when they differed by no more than 1 unit (column CN ± 1, Table 3). CBS sections also were used to average (by median) and to round copy-number assignments (column CN CBS, Table 3).

### Affymetrix SNP data

One BLC sample also was analyzed on the Genome-Wide Human SNP-array 6.0, according to the manufacturer's instructions (Affymetrix Inc., Santa Clara, CA). Normalization was performed by using the Genotyping Console™ (Affymetrix), and profile recognition was performed by using the method described in [14].

## Availability

An implementation of the proposed GAP pattern recognition and detection of copy numbers and genotypes based on segmented profiles is available, together with the supporting data [41]. SNP array data for the 19 primary tumors and the two cell lines shown here are available through Gene Expression Omnibus [37] [GEO:GSE18799].

## Abbreviations

BAF: B allele frequency; BLC: basal-like breast carcinoma; CBS: circular binary segmentation; CN: copy number; CNV: copy-number variation; FCM: flow cytometry; LOH: loss of heterozygosity; LRR: Log R ratios; mBAF: mirrored BAF; MDA_175: MDA-MB-175-VII breast cancer cell line; MDA_468: MDA-MB-468 breast cancer cell line; SNP: single-nucleotide polymorphism.

## Additional data files

The following additional data are included with the online version of this article.

A table of images of GAP patterns and copy-number recognition templates for a series of breast carcinomas with available DNA indexes (Additional data file 1), a table listing copy-number status of pericentric regions inferred on the basis of GAP pattern for a series of breast carcinomas and cell lines (Additional data file 2), two tables indicating self-consistency in copy-number attribution in dilution series calculated for two methods of recognition: GAP method and OverUnder algorithm (Additional data file 3), and GAP patterns and copy-number recognition templates for the dilution series of cell line CRL2324 (Additional data file 4).

## Authors' contributions

TP performed research, analyzed data, and wrote the paper; EM performed experiments and analyzed data; DSL designed the general project; GR provided the Affymetrix data and analyzed data; EB supervised bioinformatics analyses; and MHS designed the research and wrote the paper. All authors read and approved the final manuscript.

## Supplementary Material

Additional data file 1 A table of images of GAP patterns and copy-number recognition templates for a series of breast carcinomas with available DNA indexes Click here for file

Additional data file 2 A table listing copy-number status of pericentric regions inferred on the basis of GAP pattern for a series of breast carcinomas and cell lines Click here for file

Additional data file 3 two tables indicating self-consistency in copy-number attribution in dilution series calculated for two methods of recognition: GAP method and OverUnder algorithm Click here for file

Additional data file 4 GAP patterns and copy-number recognition templates for the dilution series of cell line CRL2324 Click here for file
