# Supplementary figures and images for: Genome Alteration Print (GAP): a tool to visualize and mine complex cancer genomic profiles obtained by SNP arrays
Source: Genome Biol. 2009 Nov 11;10(11):R128. doi: 10.1186/gb-2009-10-11-r128 (PMC2810663; doi:10.1186/gb-2009-10-11-r128)

BLC\_B1\_T17

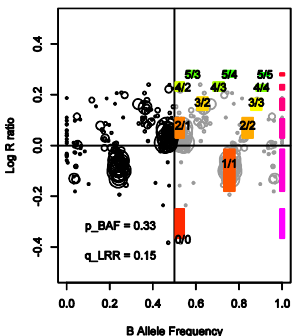

BLC\_T09

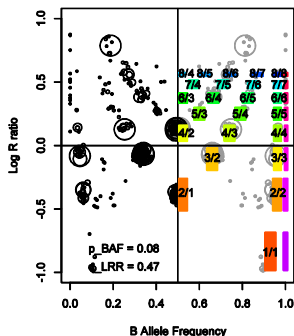

BLC\_T10

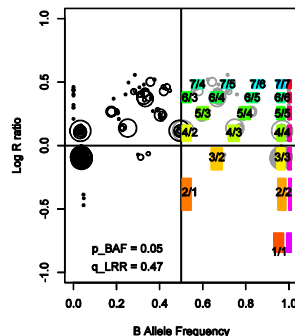

BLC\_T34

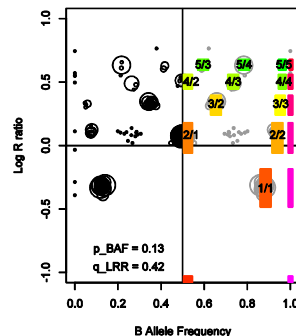

BLC\_T31

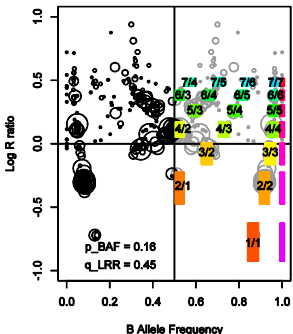

BLC\_B1\_T22

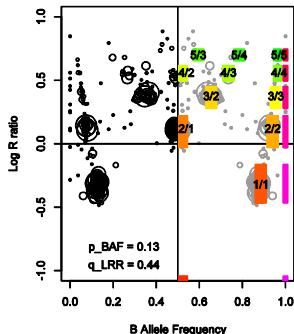

BLC\_B1\_T20

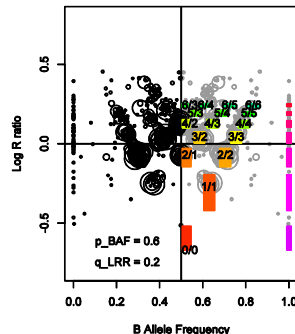

BLC\_T12

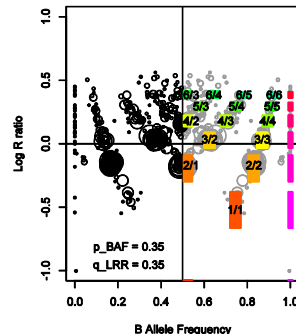

L\_B1\_T30

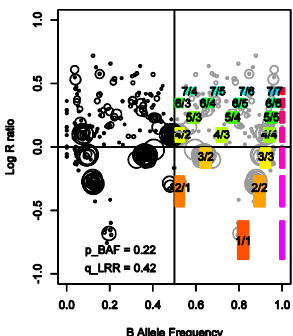

BLC\_T23

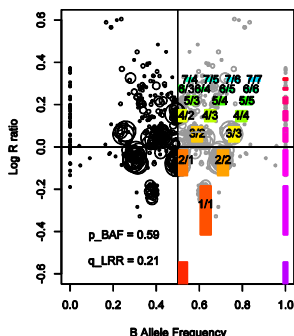

BLC\_B1\_T19

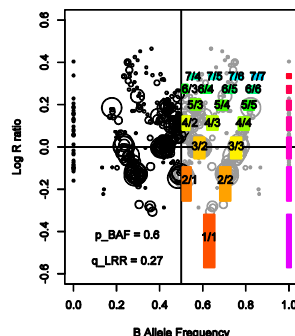

BLC\_T07

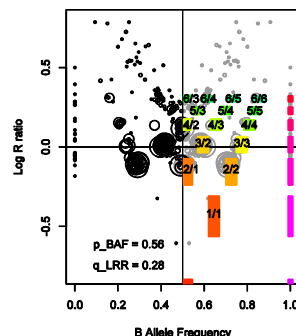

BLC\_B1\_T14

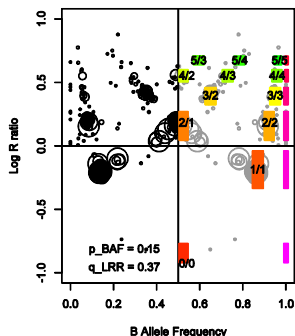

BLC\_T15

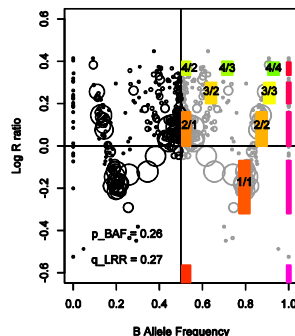

L\_B1\_T25A

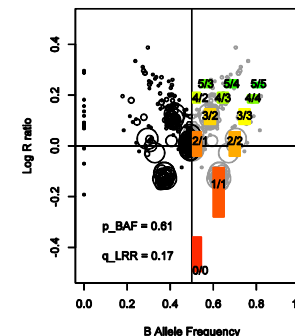

L\_B1\_T24B

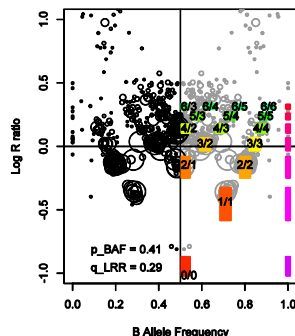

BLC\_T37

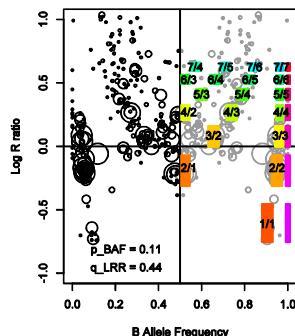

L\_B1\_T47

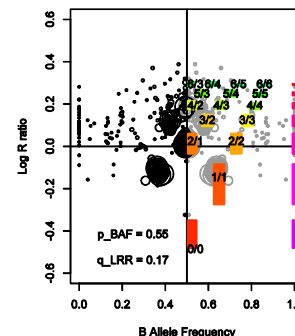

Supplement: Additional data file 1 — A table of images of GAP patterns and copy-number recognition templates for a series of breast carcinomas with available DNA indexes [file gb-2009-10-11-r128-S1.pdf]

CRL2324

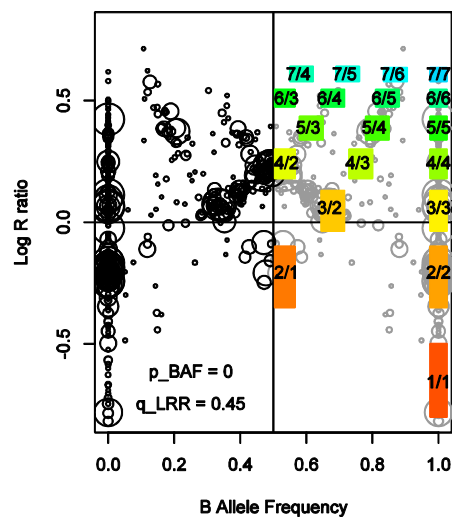

CRL2324\_79pc\_Tum

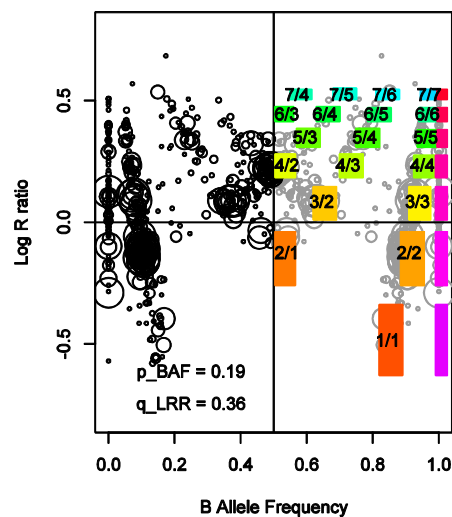

CRL2324\_50pc\_Tum

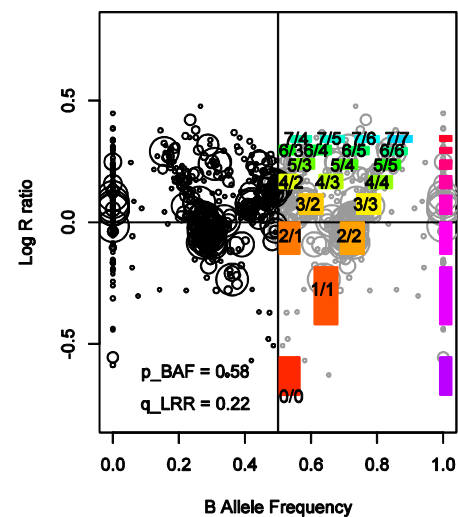

CRL2324\_47pc\_Tum

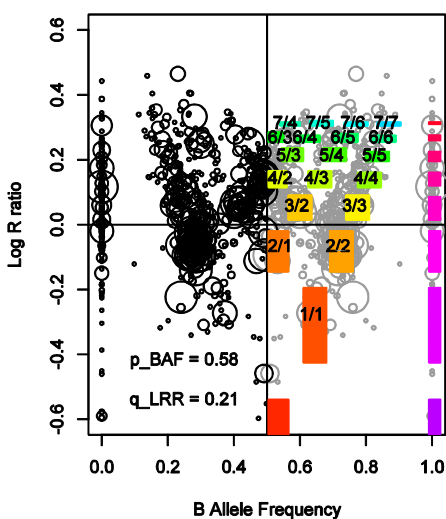

CRL2324\_45pc\_Tum

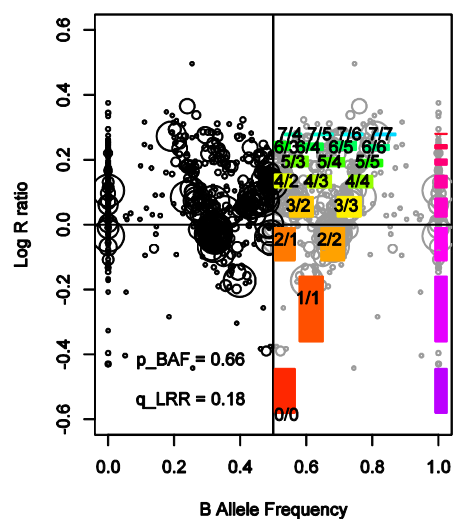

CRL2324\_34pc\_Tum

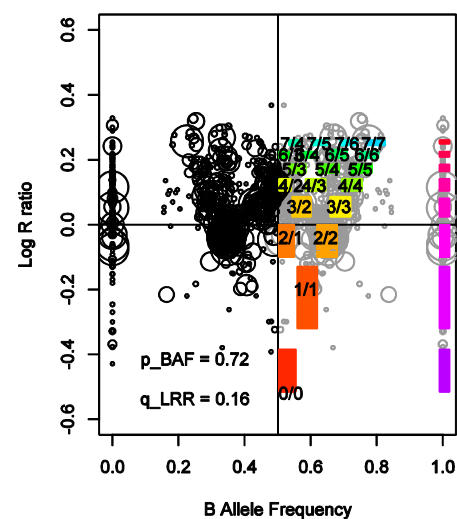

CRL2324\_30pc\_Tum

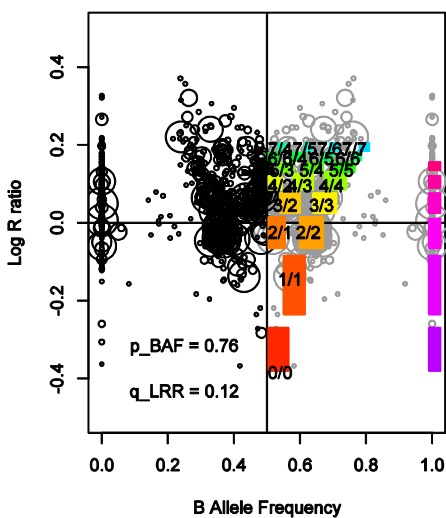

CRL2324\_23pc\_Tum

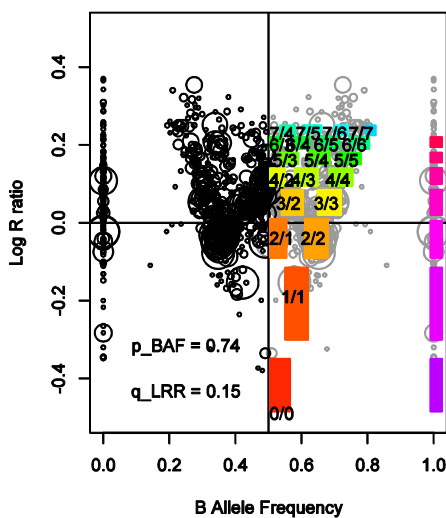

CRL2324\_21pc\_Tum

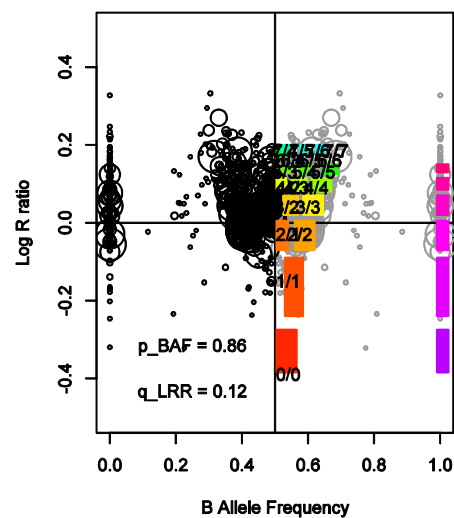

Supplement: Additional data file 4 — GAP patterns and copy-number recognition templates for the dilution series of cell line CRL2324 [file gb-2009-10-11-r128-S4.pdf]
