# Supplementary material for: Genome Alteration Print (GAP): a tool to visualize and mine complex cancer genomic profiles obtained by SNP arrays
Source: Genome Biol. 2009 Nov 11;10(11):R128. doi: 10.1186/gb-2009-10-11-r128 (PMC2810663; doi:10.1186/gb-2009-10-11-r128)
Supplement: Additional data file 3 — two tables indicating self-consistency in copy-number attribution in dilution series calculated for two methods of recognition: GAP method and OverUnder algorithm [file gb-2009-10-11-r128-S3.doc]

| OverUnder* | | Copy numbers in 79% tumor CRL2324** | | | | | | | | | | | |
| --- | --- | --- | --- | --- | --- | --- | --- | --- | --- | --- | --- | --- | --- |
|  |  | 0 | 1 | 2 | 3 | 4 | 5 | 6 | 7 | 8 | 9 | 10 | 11 |
| Copy numbers in 100% tumor CRL2324 | 0 | 1143 | 375 | 748 | 14 | 65 | 5 | 0 | 0 | 0 | 0 | 0 | 0 |
|  | 1 | 0 | 97 | 100 | 0 | 0 | 0 | 0 | 0 | 0 | 0 | 0 | 0 |
|  | 2 | 0 | 3 | 5129 | 2584 | 400 | 1088 | 7045 | 5465 | 23 | 1 | 0 | 1 |
|  | 3 | 0 | 0 | 290 | 6179 | 1179 | 1739 | 406 | 230 | 156 | 76 | 0 | 0 |
|  | 4 | 0 | 0 | 39 | 14569 | 76037 | 954 | 660 | 140 | 25 | 0 | 0 | 0 |
|  | 5 | 0 | 0 | 148 | 556 | 35462 | 8857 | 1036 | 746 | 67 | 0 | 0 | 0 |
|  | 6 | 0 | 0 | 531 | 1406 | 3827 | 44887 | 8559 | 1802 | 0 | 0 | 0 | 0 |
|  | 7 | 0 | 0 | 3447 | 292 | 305 | 15996 | 11216 | 6807 | 11 | 3 | 0 | 0 |
|  | 8 | 0 | 0 | 1105 | 306 | 81 | 1580 | 9045 | 5906 | 486 | 6 | 0 | 0 |
|  | 9 | 0 | 0 | 13 | 231 | 361 | 74 | 2077 | 6191 | 2619 | 181 | 0 | 0 |
|  | 10 | 0 | 0 | 0 | 14 | 316 | 314 | 116 | 2634 | 5556 | 440 | 86 | 0 |
|  | 11 | 0 | 0 | 0 | 9 | 273 | 577 | 172 | 154 | 1863 | 1094 | 28 | 2 |
|  | 12 | 0 | 0 | 0 | 0 | 16 | 126 | 300 | 8 | 348 | 359 | 127 | 14 |
|  | 13 | 0 | 0 | 0 | 0 | 0 | 8 | 172 | 3 | 78 | 104 | 55 | 24 |
|  | 14 | 0 | 0 | 0 | 0 | 0 | 1 | 77 | 0 | 0 | 17 | 45 | 38 |
|  | 15 | 0 | 0 | 0 | 0 | 0 | 0 | 5 | 0 | 0 | 0 | 22 | 14 |

| GAP method | | Copy numbers in 79% tumor CRL2324** | | | | | | | | |
| --- | --- | --- | --- | --- | --- | --- | --- | --- | --- | --- |
|  |  | 0 | 1 | 2 | 3 | 4 | 5 | 6 | 7 | 8 |
| Copy numbers in 100% tumor CRL2324 | 0 | 1346 | 20 | 2 | 9 | 0 | 0 | 0 | 0 | 0 |
|  | 1 | 87 | 7858 | 3263 | 36 | 0 | 0 | 0 | 0 | 0 |
|  | 2 | 29 | 791 | 132133 | 2453 | 41 | 1 | 0 | 3 | 0 |
|  | 3 | 12 | 35 | 3441 | 78811 | 1981 | 29 | 3 | 1 | 2 |
|  | 4 | 5 | 5 | 121 | 2481 | 50590 | 974 | 94 | 18 | 0 |
|  | 5 | 2 | 5 | 9 | 65 | 2271 | 19840 | 2818 | 132 | 59 |
|  | 6 | 0 | 0 | 0 | 1 | 58 | 700 | 3507 | 880 | 6 |
|  | 7 | 0 | 0 | 0 | 1 | 6 | 48 | 59 | 1586 | 10 |
|  | 8 | 0 | 0 | 0 | 0 | 0 | 2 | 0 | 3 | 54 |

*Algorithm OverUnder, Beadstudio plug-in, window length 51

**Chromosomes X, Y, were excluded from the summary because of normalization problems; chromosomes 6 and 16 were excluded because of diluting “normal” cell line CRL2325 has losses of these chromosomes.
